# Clinical management and retrieval of foreign body inclusion in a primary tooth: a case report

**DOI:** 10.1186/s13256-025-05468-9

**Published:** 2025-08-07

**Authors:** Aakriti Chandra, Nilima Thosar, Ramakrishna Yeluri, Ishani Rahate, Mrunali Deshkar

**Affiliations:** Department of Pediatric and Preventive Dentistry, Sharad Pawar Dental College and Hospital, Datta Meghe Institute of Higher Education and Research (Deemed to Be University, Sawangi (Meghe), Wardha, Maharashtra India

**Keywords:** Traumatic injury, Pediatric patient, Foreign object

## Abstract

**Background:**

While foreign body ingestion is a frequent pediatric emergency, instances of foreign objects becoming lodged in teeth are uncommon. These can lead to infections, pain, and abscesses if left untreated. Imaging techniques such as radiovisiography and cone beam computed tomography help in detection and diagnosis.

**Case presentation:**

A 6-year-old Indian girl was brought in with black discoloration in her upper front tooth (61) for 6 months. The initial history of biting a stone was inconsistent with radiographic findings. Radiovisiography showed a radiopaque object, and cone beam computed tomography confirmed a metallic foreign body—later identified as a stapler pin—embedded in the root canal. Upon further questioning, the child disclosed self-insertion of the pin. The object was retrieved, and the tooth was successfully treated with pulpectomy and strip crown cementation.

**Conclusion:**

Timely diagnosis and intervention are crucial in managing foreign body inclusions in teeth. Parents should be advised about the risks of children placing small objects in their mouths, and early treatment of carious lesions is essential.

## Background

A frequent pediatric emergency that causes great anxiety for both parents and children is the ingestion of foreign objects by accident. Toys, marbles, coins, keys, and pins are among the many objects that children often swallow. However, the intentional or accidental insertion of a foreign body into a tooth is a rare and underreported phenomenon, particularly in the pediatric population [1].

It is usually by accident that foreign things within teeth are found during radiographic or clinical tests. These foreign items can cause pulpal exposure and lodging of the foreign substance, which can lead to problems such as infection, discomfort, swelling, and recurring abscesses [2]. Frequently reported foreign objects that can irritate tissue and act as infection foci include pencil leads, metal screws, stapler pins, and toothpicks [3]. A number of radiographic methods are essential for locating and detecting foreign items inside the root canal, including triangulation, parallax views, stereo radiography, tomography, radiovisiography (RVG), and cone beam computed tomography (CBCT) scans [4]. While foreign body ingestion is a common pediatric concern, and iatrogenic introduction of objects into root canals is documented, cases detailing the self-insertion of metallic objects such as stapler pins into fractured primary incisors by young children, particularly where parental awareness is absent and initial history is misleading, are notably sparse in literature. This case report aims to document such an unusual instance, detailing the diagnostic pathway that navigated an initially unclear history, the crucial role of the child’s delayed disclosure, and the utility of CBCT in confirming the diagnosis and guiding safe removal when conventional radiography was equivocal, and to underscore the clinical vigilance required in managing pediatric dental trauma with unusual sequelae.

## Case presentation

**Fig. 1:**
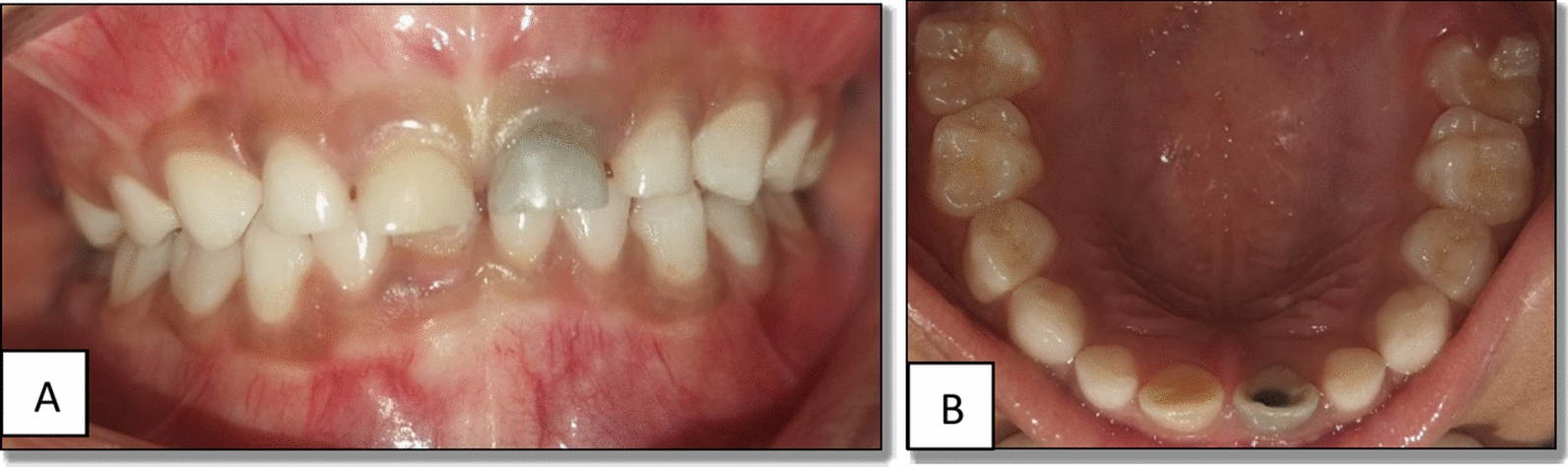
Nonvital primary tooth (61) after trauma with an open pulp chamber; **A** labial view; **B** occlusal view

**Fig. 2:**
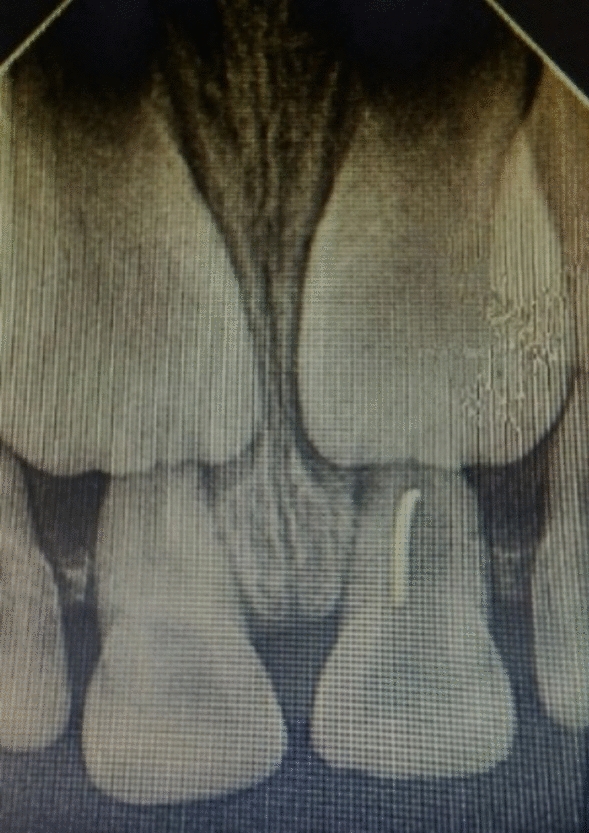
Radiovisiography of primary tooth (61)

**Fig. 3:**
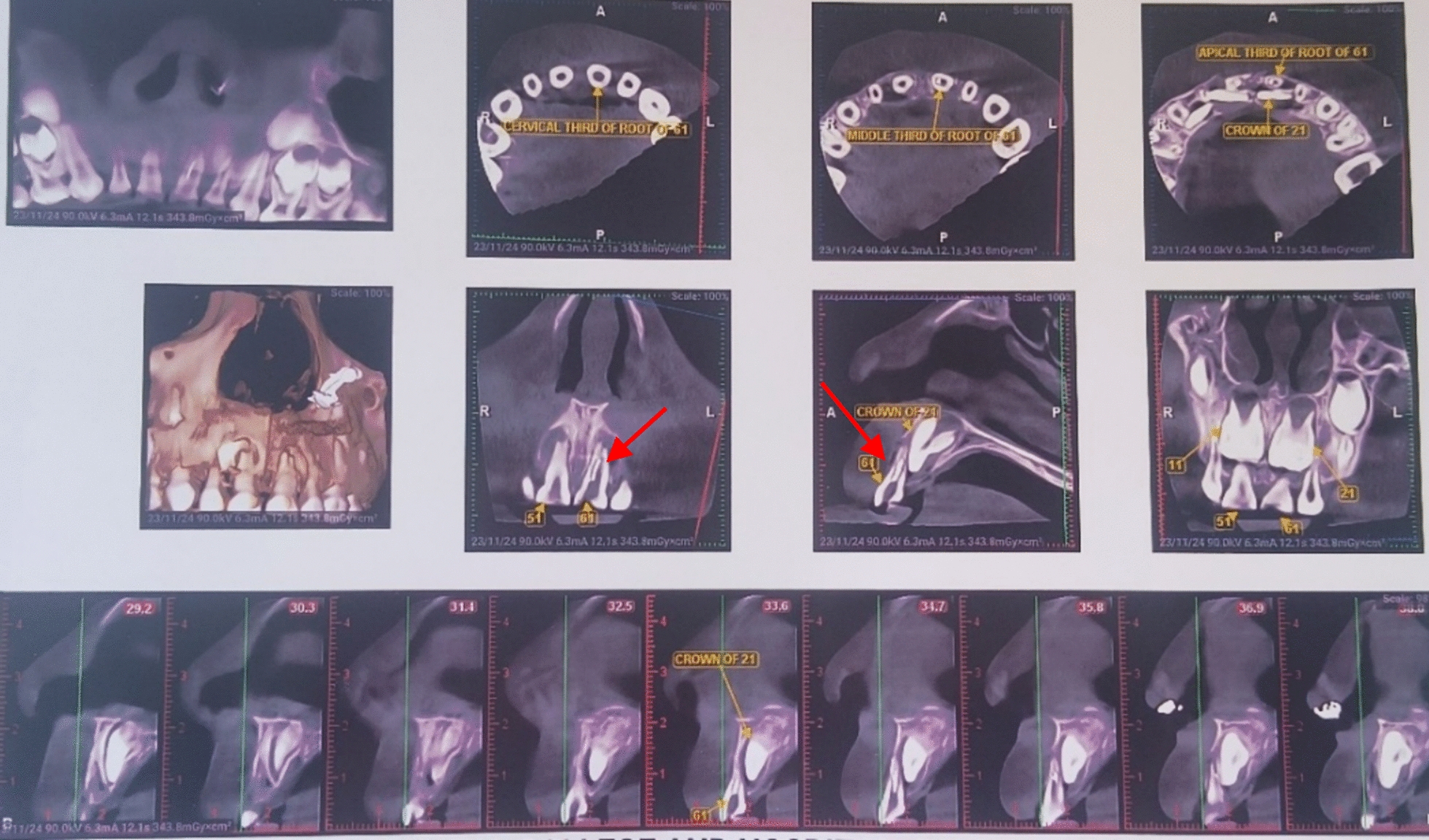
Cone beam computed tomography of tooth 61 with red arrow confirming the location of foreign object in the primary tooth

A 6-year-old Indian female patient presented to the department with her parents with a complaint of blackish discoloration in the upper front tooth region of the jaw for the past 6 months. Parents also provided a history of biting a stone from that region about a year ago, but were unaware. No other significant systemic medical conditions, allergies, or relevant past medical or family history were reported by the parents. The child had visited a private dental clinic for the same issue but did not receive any intervention. The clinical examination revealed a blackish discoloration on the left deciduous central incisor, or tooth 61, with an open pulp chamber with lodgement of food debris (Fig. 1). A previously obtained radiovisiography (RVG) from a private dental clinic showed a linear radiopaque line in the pulp chamber (Fig. 2). Since history provided by the parents did not suggest the possibility of a foreign body, the RVG finding was initially interpreted as a possible artifact. Given the unclear nature of the radiograph and the absence of corroborating history, there was a critical need for precise three-dimensional localization of the suspected foreign body to ensure its safe retrieval, minimize trauma to the tooth and surrounding structures, including the developing permanent successor, and differentiate it from an artifact. Cone beam computed tomography (CBCT) scan was deemed necessary. Informed consent was acquired from the child’s parents after a thorough explanation of the procedure to identify the precise position and extent of a suspected foreign substance within tooth 61’s root canal that provides a three-dimensional image for accurate evaluation and treatment planning. They were also informed about the procedure, radiation exposure, potential risks, benefits, and alternative diagnostic methods. Assurance was given that the procedure would follow pediatric safety norms with minimal radiation exposure. CBCT of tooth 61 revealed similar results, showing vertical linear opacity seen in the middle third of the root canal, suggestive of a metallic foreign body, thus confirming the diagnosis and locating the exact position of the foreign object (Fig. 3).

Following this confirmation, further gentle and empathetic questioning was conducted with the child, away from immediate parental presence. In this supportive setting, the child disclosed a previously unrevealed habit of placing stapler pins in her mouth. She admitted that she had inserted a stapler pin into the fractured opening of tooth 61. This critical piece of information clarified the true sequence of events, making it evident that the stone-biting incident was unrelated to the foreign body insertion.

**Fig. 4:**
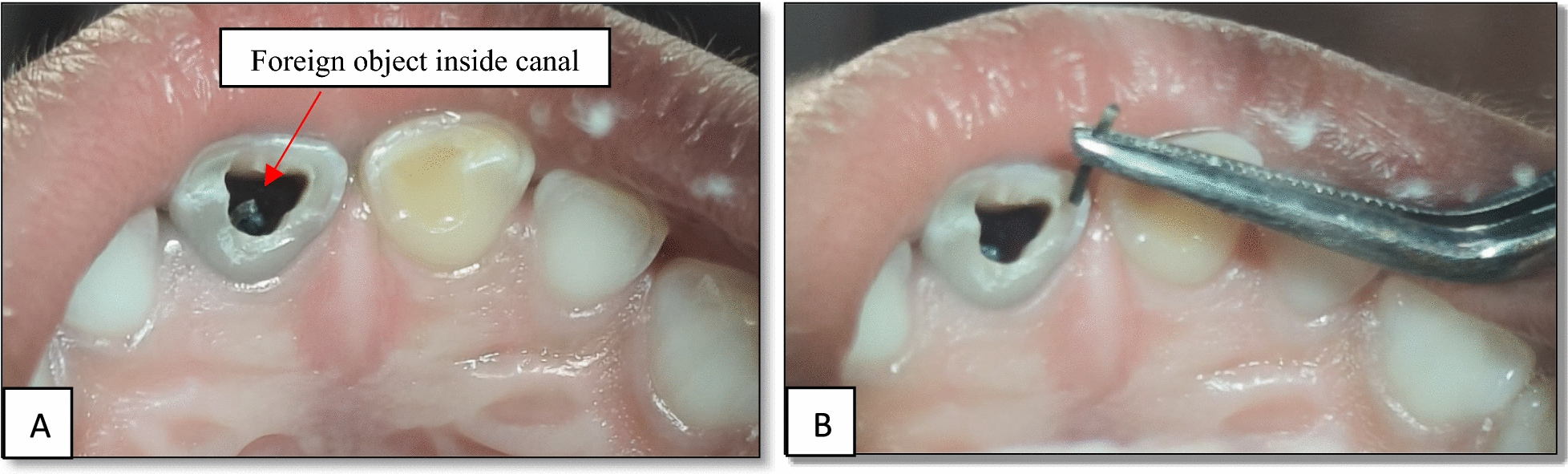
Removal of foreign object from the pulp canal; **A** foreign object inside a canal; **B** retrieval of foreign object

**Fig. 5:**
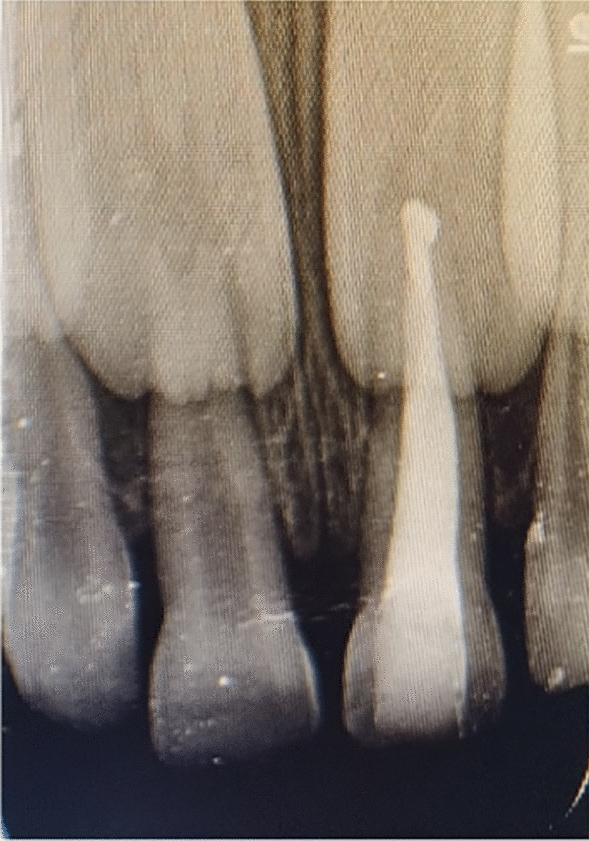
Postoperative radiograph of tooth 61

**Fig. 6:**
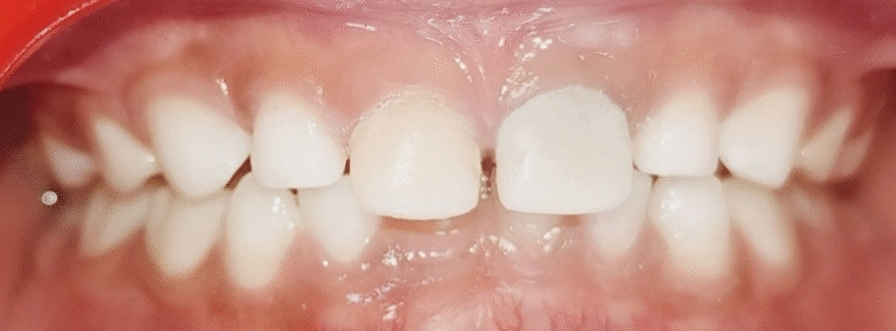
Postoperative clinical image of the case after strip crown cementation

**Fig. 7:**
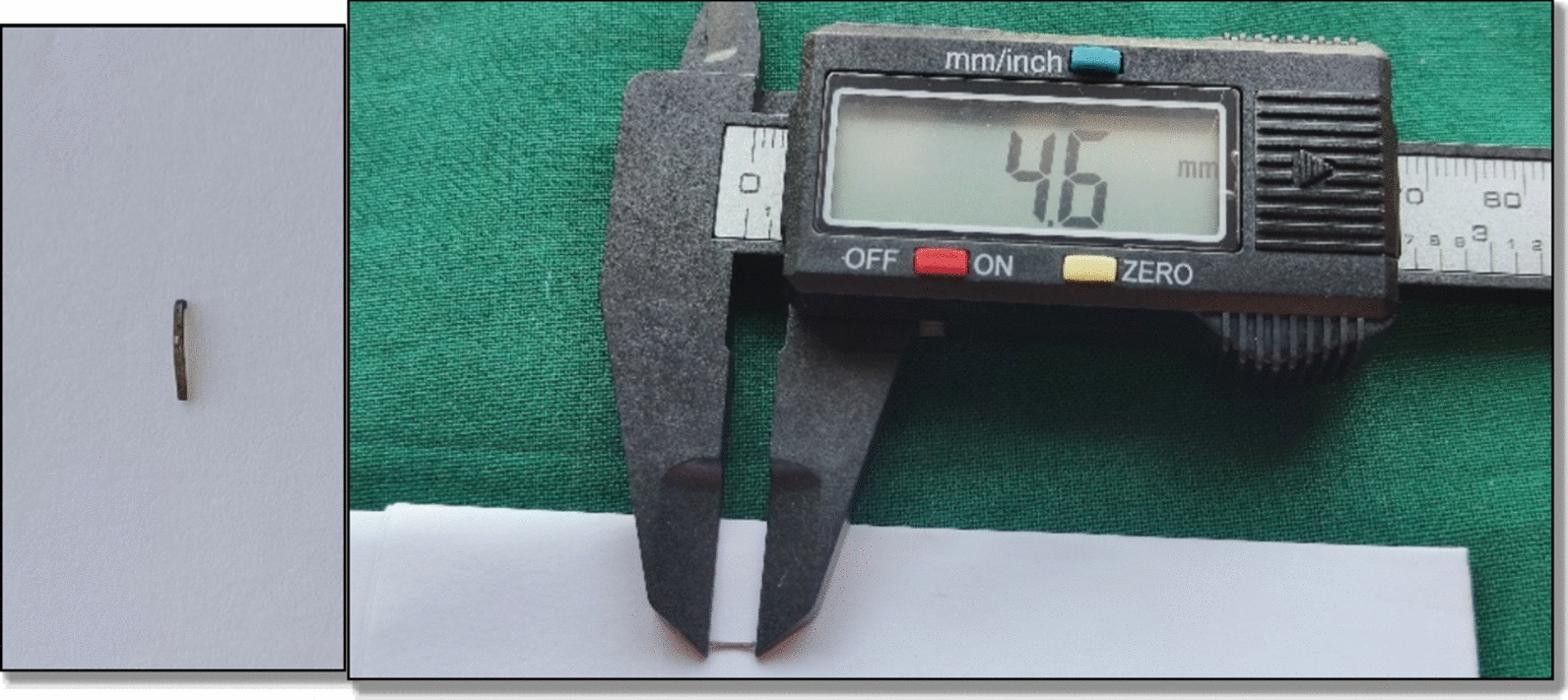
The stapler pin, out of the canal, measures about 4.6 mm

Considering the age of the patient and root length of the teeth, it was planned to save the tooth by retrieving the foreign object from the pulp canal. Following parental consent and local anesthesia, attempts were made to isolate tooth 61 using a rubber dam. However, owing to the patient’s young age and limited cooperation, achieving effective rubber dam isolation was not feasible. Consequently, the treatment was performed under meticulous cotton roll isolation with high-volume evacuation. Access to the tooth was made, and using an H-file, the foreign object was retrieved (Fig. 4). The tooth was subsequently treated further for pulpectomy (Fig. 5), followed by strip crown cementation (Fig. 6). Upon further examination, the foreign object was identified as a stapler pin of about 4.6 mm (Fig. 7). The case was further evaluated after 1 month for follow-up, revealing that no periapical radiolucency was detected.

## Discussion

Children often place objects in their mouths, leading to risks of accidental ingestion or aspiration, which can distress parents and pose significant dangers if the objects are pointy or sharp. Such incidents might result in severe complications, including esophageal tears, carotid artery rupture, and cardiac tamponade [5, 6]. Foreign bodies inside teeth can act as infection sources, causing pain, swelling, and bleeding. Rare cases include jewellery lodged in a maxillary incisor, leading to actinomycosis [7]. Reports by Grossman, Gelfman, and Harris document the retrieval of various items, such as crayons, bristles of toothbrushes, toothpicks, pencil points, and pins from anterior teeth with open root canals [8–10].

It is uncommon for items to be inserted into teeth, and youngsters often conceal this from their parents out of fear. In this case, the parents were unaware that the child had inserted a stapler pin into her mouth, and the child did not disclose this habit during the initial history-taking. They were appalled when the pin was seen on the radiograph. Pain, bleeding, and swelling can be caused by foreign objects inside the tooth that serve as an infection focal point.

Obtaining an accurate history in pediatric dental cases often presents a challenge owing to children’s limited communication skills, fear of reprimand, or deliberate concealment of potentially blameworthy behaviors. In this case, the initial parental report of a stone-biting incident, though noted, appeared insufficient to explain the presence of a metallic object deeply embedded within the pulp canal. This initial account was thus implausible as a complete etiological explanation. The turning point in diagnosis came after CBCT imaging confirmed the presence of a metallic foreign body. This prompted a more sensitive and empathetic approach in questioning the child, away from parental pressure, which led to the eventual disclosure of her habit of placing stapler pins in her mouth and specifically into the fractured tooth. This sequence—starting from a misleading history, followed by radiographic clarification, and concluding with the child’s confession—illustrates a common behavioral pattern in pediatric patients where fear or shame may suppress accurate reporting. The case highlights the importance of establishing a supportive clinical environment that facilitates open communication and helps bridge the gap between clinical findings and patient history.

Several foreign objects have been documented as being lodged in teeth. For example, Toida documented an unerupted supernumerary tooth with an embedded plastic chopstick in a 12-year-old boy. Other cases, such as those cited by Zillich and Pickens, describe hat pins and dressmaker pins breaking inside the root canals of incisors during endodontic treatment [9]. Similarly, Kanumuri *et al.* retrieved multiple metal wires and a stapler pin from an infected primary molar in a 10-year-old child, with the foreign body likely inserted repeatedly into a wide carious lesion [2]. These cases share common features with the present report, including the affected age group (2–10 years) and the maxillary anterior teeth being the most commonly involved. Stapler pins, as in this case, are frequently reported foreign objects owing to their accessibility and size. However, unlike Kanumuri’s case, the current report involves a single object in a deciduous maxillary central incisor without a clear procedural origin, suggesting an intentional or exploratory insertion by the child. This distinction is important, as it shifts the focus from iatrogenic or treatment-related causes to behavioral tendencies and the role of patient history in diagnosis. Foreign bodies can be ingested and positioned inside a bodily cavity or deposited through traumatic or iatrogenic injuries, often resulting in abscess formation, septicemia, or hemorrhage. Common iatrogenic lesions include the deposition of endodontic materials, amalgam tattoos, and other dental materials introduced during trauma or treatment [11, 12].

Foreign bodies in teeth are most common in children aged 2–20 years, with maxillary central incisors being the most frequently affected. Stapler pins are the most commonly found objects, and retrieval success depends on the location of the object relative to the curvature of the canal [5]. However, the present case differs markedly in several aspects. Unlike the abovementioned examples, this report involves the self-insertion of a single stapler pin into a primary maxillary incisor, with no evident history of previous dental intervention or ongoing infection that might have prompted repeated insertion. The object was not introduced during or following a clinical procedure but rather was placed by the child herself, likely as a result of exploratory behavior or curiosity. Critically, neither the parents nor the child initially disclosed this behavior. The parents attributed the discoloration to an earlier episode of trauma from stone biting, and the child did not admit to inserting a pin until prompted after radiographic confirmation. This behavioral concealment aligns with literature noting children’s fear or shame in disclosing such actions but highlights the diagnostic challenge of relying solely on history in pediatric cases.

Furthermore, the use of imaging played a pivotal role in confirming the diagnosis. While initial radiovisiography (RVG) showed a vague radiopaque line within the root canal, it was inconclusive in terms of object identification and precise localization. A CBCT scan was subsequently employed, revealing a vertically oriented foreign body in the mid-root canal. However, the use of CBCT offered a more precise three-dimensional assessment of the object’s position and its relationship to the surrounding structures. This enhanced imaging allowed for precise localization, improved planning of the retrieval procedure, and minimization of trauma to the developing permanent tooth bud. Despite concerns regarding radiation exposure in pediatric populations, this case justifies the selective use of CBCT when conventional imaging and patient history are insufficient or conflicting. The scan enabled accurate diagnosis, assessment of the object’s spatial orientation, and planning of a conservative retrieval approach, thereby preserving the tooth and preventing damage to the underlying permanent successor.

This case offers several novel insights that reinforce the importance of a holistic diagnostic approach in pediatric dentistry:

- It emphasizes the behavioral dimension of self-insertion of foreign objects, especially in cases where there is a delay or failure in disclosure by both the child and parents.
- It demonstrates the limitations of relying solely on two-dimensional radiography and patient history, and supports the judicious use of CBCT in complex pediatric diagnostic scenarios.
- It contributes to the sparse literature on foreign body insertion into primary incisors, particularly with a noniatrogenic origin and behavioral causation, expanding our understanding of pediatric patient behavior and risk factors.

In this case, with parental consent, the foreign object was extracted to prevent complications, aligning with similar treatments reported in literature [13]. Effective management of such cases requires timely diagnosis and intervention to avoid further issues. While rubber dam isolation is the standard of care in endodontic procedures, its use in uncooperative pediatric patients can be difficult. In this case, owing to limited cooperation, cotton roll isolation with high-volume suction was used to maintain asepsis. This reflects the balance between ideal practice and practical challenges in pediatric dentistry. From a clinical perspective, this case highlights the importance of early intervention in carious lesions, which can otherwise serve as portals for foreign body insertion. This case utilities CBCT imaging selectively when RVG results are inconclusive, and the clinical presentation suggests an underlying abnormality, despite the associated radiation exposure, particularly when it can aid in preserving the primary tooth and avoiding injury to the permanent successor. It also underscores the need for parental awareness and education regarding the risks of unsupervised access to small objects and the importance of routine dental check-ups. Pediatricians and dentists should counsel parents to monitor children’s behaviors closely and seek timely dental care when caries or trauma is present. Thus, adopting a nonjudgmental and child-centered approach during history-taking is crucial, fostering an environment in which children feel safe to disclose potentially embarrassing or fear-inducing behaviors.

## Conclusion

This case contributes meaningful insight into the diagnostic, behavioral, and preventive aspects of foreign body insertion in primary teeth. It also illustrates the judicious use of advanced imaging such as CBCT when standard methods are insufficient, reinforcing the importance of individualized diagnostic planning in pediatric dental care. Further, parents should also be advised to keep small objects out of their children’s reach to avoid such emergencies. It is also important to manage open carious lesions early and encourage a nonjudgmental, communicative approach with children. Such instances should be diagnosed using a comprehensive history, a competent clinical examination, and appropriate imaging of the suspicious location.

## Data Availability

Not applicable.

## Abbreviations

RVG: Radiovisiography
CBCT: Cone beam computed tomography
